# *Allium sativum* L. var. Voghiera Reduces Aflatoxin B1 Bioaccessibility and Cytotoxicity *In Vitro*

**DOI:** 10.3390/foods13030487

**Published:** 2024-02-02

**Authors:** Álvaro Lázaro, Massimo Frangiamone, Annalisa Maietti, Alessandra Cimbalo, Pilar Vila-Donat, Lara Manyes

**Affiliations:** 1 Laboratory of Food Chemistry and Toxicology, Faculty of Pharmacy and Food Sciences, University of Valencia, 46100 Burjassot, Spain; alvaro.lazaro@uv.es (Á.L.); massimo2.frangiamone@uv.es (M.F.); pilar.vila@uv.es (P.V.-D.); lara.manyes@uv.es (L.M.); 2 Department of Chemical, Pharmaceutical and Agricultural Science, University of Ferrara, Via Luigi Borsari 46, 44121 Ferrara, Italy; annalisa.maietti@unife.it

**Keywords:** mycotoxin, garlic, prebiotic, Jurkat T cells, *in vitro* digestion, flow cytometry

## Abstract

The present work focuses on the evaluation of AFB1′s bioaccessibility and cytotoxicity *in vitro* using bread (naturally contaminated) enriched or not enriched with fresh Voghiera garlic (2%). Two different experiments were carried out: experiment 1 (E1), with low-AFB1-concentration breads (1.6–1.7 mg/kg); and experiment 2 (E2), with high-AFB1-concentration breads (96.4–102.7 mg/kg). Eight breads were prepared, four for E1 (experiment 1) and another four for E2 (experiment 2), with each experiment having a control group (C), a garlic-enriched group (2%) (G), an AFB1 group (A), and an AFB1 + garlic group (A + G). Simulated digestion was performed on each type of bread, and gastric and intestinal digests were obtained. AFB1 content in flours, baked bread, and gastric and intestinal digests was measured by High-Performance Liquid Chromatography coupled to Fluorescence Detection. The results demonstrate dose-dependent AFB1 bioaccessibility and that the presence of garlic contributed to its reduction in both doses (7–8%). Moreover, garlic’s presence in AFB1-contaminated bread increased cell viability (9–18%) in differentiated Caco-2 cells and mitigated the arrest of S and G2/M phases provoked by AFB1 on Jurkat T cells and reduced apoptosis/necrosis, cellular reactive oxygen species (ROS), and mitochondrial ROS by 16%, 71%, and 24% respectively. The inclusion of garlic as a functional ingredient helped relieve the presence and effects of AFB1.

## 1. Introduction

Aflatoxins (AFs) are secondary metabolites produced by mycotoxigenic fungi belonging to *Aspergillus* genera which contaminate a wide variety of crops, such as cereals, oilseeds, tree nuts, and spices. Among them, aflatoxin B1 (AFB1) is the most studied due to its severe carcinogenicity for humans and animals [1]. In fact, the International Agency for Research on Cancer classified AFB1 as a human carcinogen belonging to Group 1 due to its ability to form DNA adducts [2]. Different *in vitro* and *in vivo* studies have demonstrated its acute and chronic toxicity, which can lead to the onset of liver damage, nutritional dysfunctions, immunosuppression, and cancer [3,4,5].

As mycotoxins are major contaminants of cereals, it has been found that AFB1 contaminates feeds universally [6]. It has been shown that in 87.5% of samples found to contain AFB1, its level was higher than the allowed level, which was 2 μg/kg from 2006 to 2023 [7]. Despite treatments applied during the production process, the natural occurrence of this mycotoxin has been observed in commercial bread loaves [8], as well as in maize and in wheat bread [9,10]. Consequently, to enhance AFB1 reduction in bakery products, the use of bio-preservatives in their formulation could be a good alternative to conventional procedures [11]. Following this trend, the addition of functional ingredients from various food matrices rich in bioactive compounds has been previously investigated to assess AFB1 bioaccessibility reduction through simulated gastrointestinal digestion models [12,13,14,15,16]. Likewise, the cytoprotective effect of some natural compounds against mycotoxin was demonstrated by using different cell models [17,18].

Among others, garlic plant (*Allium sativum* L.) has been shown to exert multiple health benefits, such as the prevention and treatment of cardiovascular diseases, antioxidant activity, and antimicrobial effects [19]. The variety “Voghiera” is a typical Italian eco-type of garlic cultivated in a particular area north-east of Voghiera (Ferrara) in the Emilia-Romagna region and classified as a Protected Designation of Origin product. Phytochemical analysis of this garlic has been used for the isolation of several saponins, such as furostanol, voghierosides (A1, A2, E1, E2), spirostanol, and eugenol diglycosides, which possess important antimicrobial activities [20]. In addition, its role in mitigating the evolution of cancer progression was recently investigated [21].

Thus, the aim of this study was to evaluate the possible beneficial effect of Voghiera garlic on bread in terms of modifying AFB1′s bioaccessibility and cytotoxicity by using Caco-2 cells, which are widely used as a prototype of the intestinal epithelial barrier [22], and Jurkat T human cells, which are also broadly used in *in vitro* studies of T cytokines, receptor expression, and cell signal transduction lines [23].

## 2. Materials and Methods

### 2.1. Reagents

AFB1 standard solution (purity > 99%) was acquired from Sigma-Aldrich (St. Louis, MO, USA). Potassium thiocyanate (KSCN), potassium chloride (KCl), hydrochloric acid (HCl), sodium dihydrogen phosphate (NaH_2_PO_4_), sodium hydroxide (NaOH), sodium sulfate (Na_2_SO_4_), sodium chloride (NaCl), sodium hydrogen carbonate (Na-HCO_3_), urea (CO(NH_2_)_2_), pepsin A (674 U mg^−1^ P7000), pancreatin (762 U mg^−1^ P1750), bile salts (B8631), and α-amylase (930 U mg^−1^ A3403) were purchased from Sigma-Aldrich (Madrid, Spain). Acetonitrile (ACN) and methanol (MeOH) were obtained from Fisher Scientific (Madrid, Spain). Deionized water was obtained from a Milli-Q water purification system (Millipore, Bedford, MA, USA). Dimethyl sulfoxide (DMSO) was acquired from Fisher Scientific (Geel, Belgium). Bread ingredients were purchased at a local supermarket. Fresh garlic was preserved at 4 °C and minced before use. Propidium iodide (PI) and a cycleTEST™ PLUS DNA Reagent Kit were obtained from BD Biosciences (San Diego, CA, USA) for the cell cycle assay by flow cytometry. The Annexin V–FITC conjugate was acquired from Miltenyi and the MitoTracker^®^ probe was purchased from Invitrogen (Waltham, MA, USA). 2′,7′-dichlorodihydrofluorescein diacetate (H_2_-DCFDA) and phosphate buffer saline (PBS) were bought from Sigma Chemical Co. (St. Louis, MO, USA).

### 2.2. Maize Flour Contamination and Mycotoxin Production

Maize was contaminated by *A. flavus ITEM 8111* acquired from the Agro-Food Micro-bial Culture Collection of the Institute of Sciences and Food Production (ISPA, Bari, Italy). The fungus was kept under ideal conditions in the laboratory to produce the mycotoxin in the food matrix (maize) in which it was inoculated. Under these conditions, the strain used only generated this mycotoxin and not any others. A total of 450 g of maize was introduced per 1 L previously sterilized jar, and two jars were prepared. The contamination was carried out by adding 20 mL of mycelium suspension and spores in peptone water with Tween 80 of the fungal strain. Glass jars were maintained at room temperature for a period of three weeks. Every 2–3 days, 1 mL of distilled water was added to the jars to maintain the humidity conditions.

After three weeks, the jars were autoclaved to eliminate fungal contamination and the maize was transformed into flour until total homogenization was achieved, including sieving. The maize samples were kept at −20 °C [12]. The maize flour was analyzed via High-Performance Liquid Chromatography coupled to Fluorescence Detection (HPLC-FLD) (as reported in Section 2.6) to quantify AFB1 and discard any other mycotoxins.

### 2.3. Bread Ingredients, Bread Making, and Baking

Eight breads were made combining the bioactive ingredient and the AFB1, four corresponding to experiment 1 (E1) and another four corresponding to experiment 2 (E2): a control bread (C), 2% garlic bread (G), AFB1 bread (A), and AFB1 + 2% garlic bread (A + G). Control bread was prepared using the following recipe: 127 g of wheat flour, 66 mL of mineral water (37 °C), 8 g of yeast for bakery products (Levital, Sant Hilari Sacalm, Spain), 4 g of sugar, and 2.6 g of NaCl. After merging all the ingredients, homogenization was conducted in a SilverCrest Bread Maker SBB 850 A1 (Kompernass GMBH, Bo-chum, Germany) for 5 min, and they were shaped in molds (approximately 200 g), covered using a damp cloth, and fermented for 1 h at room temperature. Afterwards, the breads were covered with silver foil and baked at 180 °C for 35 min in a Memmert ULE 500 muffle furnace (Madrid, Spain). Then, the breads were unmolded and refrigerated at room temperature for 1 h. Enriched breads were prepared by slightly modifying the control recipe to produce 2% garlic bread (G and A + G). Finally, breads contaminated with AFB1 were prepared by replacing some of the wheat flour with 5.9 g (E1~152 mg/kg) and 54.7 g (E2~197 mg/kg) of maize flour contaminated with AFB1 to obtain low-concentration and high-concentration breads, respectively. This difference in AFB1 concentration was used to determine if it played an important role in bioaccessibility. As shown in Table 1, eight breads were obtained in total.

### 2.4. In Vitro Static Digestion Model

The digestion process consisted of three phases: (oral, gastric, and intestinal), as re-ported in previous work [12]. In short, 10 g of bread was put in sterilized plastic bags (500 mL) and mixed with Milli-Q water (at 37 °C) and 6 mL of artificial saliva (Millipore, Bedford, MA, USA). To reproduce the oral phase, an IUL Stomacher (IUL S.A, Barcelona, Spain) was used for 30 s to simulate the mastication process. Saliva was prepared the day before (and adjusted to pH = 6.8) by mixing 0.17 mL of NaCl (175.3 g/L), 1 mL of KSCN (20 g/L), 1 mL of NaH_2_PO_4_ (88.8 g/L), 2 mL of NaHCO_3_ (84.7 g/L), 1 mL of KCl (89.6 g/L), 1 mL of Na_2_SO_4_ (57 g/L), 29 mg of α-amylase, 0.8 mL of urea (25 g/L), and distilled water to achieve a volume of 100 mL. Once the oral phase was simulated, the content was transferred to a topaz Erlenmeyer flask to carry out the gastric phase. The mixture was acidified to pH = 2 with 6 N HCl solution. Then, 14 mL of Milli-Q water (at 37 °C) and 0.5 g of pepsin solution (1 g in 25 mL of 0.1 N HCl) were added to achieve 100 mL. Samples were incubated for 2 h at 37 °C under darkness and slight agitation (1 g) using an orbital shaker (Infors AG CH-4103, Bottmingen, Switzerland). Following that, gastric aliquots (15 mL) were kept for further analysis and pancreatic digestion was conducted by adding 1.25 g of a bile salt/pancreatin mixture (0.625 g of bile salts and of 0.1 g of pancreatin dissolved in 25 mL of 0.1 N NaHCO_3_) at pH = 6.5 (0.5 N NaHCO_3_). Digests were incubated as formerly detailed (2 h at 37 °C in darkness and slight agitation), the pH was finally adjusted to 7.2 (0.5 N NaOH), the samples were centrifuged (226× *g* for 10 min at 4 °C), and the supernatant was gathered to obtain the intestinal digests. The experiments were performed in triplicate and quadruplicate.

### 2.5. AFB1 Extraction from Bread and Digest Samples

A total of 5 g of maize/wheat/bread flour was placed in centrifuge tubes (50 mL). Then, 25 mL of MeOH/H_2_O (80/20) was added. Ultraturrax (T 18 digital ULTRA-TURRAX^®^, Staufen, Germany) was used for 3 min to crush the samples after being centrifuged at 226× *g* for 5 min (Centrifuge 5810R, Eppendorf, Germany). The supernatant was collected and filtered through a 0.22 μm filter (Phenomenex, Madrid, Spain). All analyses were performed in triplicate (*n* = 3). Gastric and intestinal digest samples were centrifuged at 226× *g* for 10 min at 4 °C, filtered (0.22 μm filter) (Phenomenex, Madrid, Spain), diluted with MeOH (1:1), and injected in HPLC-FLD.

### 2.6. Quantitative Determination of AFB1 by HPLC-FLD

AFB1 was characterized using HPLC with an Agilent 1100 series (Agilent Technologies, Santa Clara, CA, USA) furnished with an automatic sampler, a degasser, a quaternary pump, and an FLD detector Agilent 1200 (Agilent Technologies, Santa Clara, CA, USA), and Agilent Software JP03924119 was used for data analysis.

A derivatization system (UVE™ Photochemical reactor, LCTech, Jasco Analitica, Madrid, Spain, S.L.) was placed between the analytical column and the FLD detector to improve the fluorescent activity of AFB1. The chromatographic separation was carried out by using a reverse phase column, 00F-4633-EO Kinetex EVO C18 (150 × 4.6 mm, 100 A, and particle size of 5 µm) (Phenomenex, Palo Alto, CA, USA) at 40 °C. The isocratic mobile phase was composed of H_2_O/ACN/MeOH (60:10:30 *v*/*v*/*v*) with a flow rate of 1 mL min ^1^. The injection volume was 20 µL for flour and breads and 40 µL for gastrointestinal digests. The AFB1 excitation and emission wavelengths were λex = 365 nm and λem = 440 nm.

### 2.7. Quantitative Determination Method Validation

Linearity was assessed by means of the coefficient of regression (r^2^) of calibration matrix-matched curves. The limit of detection (LOD) and limit of quantification (LOQ) were measured through a comparison of the chromatographic signals from samples containing low concentrations of the compound to those of blank samples and establishing the minimum concentration at which the compound can be accurately detected and measured, respectively. Signal-to-noise ratios of 10:1 and 3:1 was considered to obtain the LOD and LOQ, respectively. Repeatability and intermediate precision were quantified as the relative standard deviation of four analyses performed on four different days (*n* = 4) and four analyses performed on the same day (*n* = 4), respectively.

Flour and bread matrix-matched curves were prepared by fortifying, with an AFB1 standard (100 mg/L in MeOH), the extracted solution from the blank (bread with uncontaminated wheat flour) with different AFB1 concentrations to obtain at least 6 level point curves. Validation parameters for the AFB1 quantitative determination method (linear regression equation, linearity range, r^2^, LOD/LOQ) are described in Table 2.

Matrix-matched calibration curves were obtained by spiking blank digest (gastric and intestinal) with the AFB1 standard (10 mg/L in MeOH) at different concentrations to obtain at least 6 level point curves (Table 2).

### 2.8. Bioaccessibility Study

Bioaccessibility was calculated for the gastric and intestinal phases. The calculation of AFB1 bioaccessibility (%) was considered as the percentage of mycotoxin from the initial bread that was detected in the intestinal digests. The quantity of mycotoxin (μg) in 10 g of bread was obtained from bread concentration (μg/kg) by conversion factors (×10/1000). The quantity of mycotoxin (μg) in 100 mL of digest was obtained from digest concentration (μg/L) by conversion factors (×100/1000), as can be seen in the formula below.

Bioaccessibility = digest concentration (μg/L) × 1000/bread concentration (μg/kg)

### 2.9. Cell Culture

Human colorectal adenocarcinoma cells, Caco-2 (ATCC HB-8065), were kept in DMEM supplemented with 10% fetal bovine serum (FBS), 100 mg/mL streptomycin, and 100 U/mL penicillin. To culture cells, 75 cm^2^ plastic flasks with filter screw caps kept in a in an atmosphere containing 5% CO_2_ at 95% relative humidity and 37 °C were used. A specific number of sub-passages (from 15 to 20 passages) were habitually controlled to maintain genetic homogeneity. The culture medium was changed every two days.

Jurkat T cells (ATCC-TIB152) were kept in RPMI 1640 medium supplemented with 10% FBS, 100 mg/mL streptomycin, and 100 U/mL penicillin. The conditions of incubation were pH = 7.4, 37 °C under 5% CO_2_, and 95% constant humidity. A small number of sub-passages (from 25 to 30 passages) were habitually controlled to maintain genetic homogeneity. The culture medium was changed every two days.

### 2.10. Cell Viability Assay

Caco-2 cells were cultured in 24-well tissue culture plates by adding 500 μL/well of a suspension of 2 × 10^6^ cells/mL. Twelve plates were needed, four for each group for three iterations each (24, 48, and 72 h). Every 3 days, the medium was replaced. After 21 days, which is the time necessary for Caco-2 cells to differentiate, the exposure was carried out. The exposure consisted of serial dilutions (non-diluted, ½, ¼, 1/8, 1/16, and 1/32) of intestinal digests from E2, since it contained the optimal AFB1 concentration needed to conduct the experiments in contrast to E1, whose AFB1 levels were very low. Four wells per condition were tested.

To determine the viability of cells, the 3-[4,5-dimethylthiazol-2-yl]-2,5 diphenyl tetrazolium bromide (MTT) assay was used. This consists of the reduction of yellow soluble tetrazolium salt to an insoluble purple formazan crystal through a reaction independent of the mitochondria. Briefly, after exposure studies with the intestinal digests (24, 48, and 72 h), the medium containing these compounds was eliminated, and the cells of each well-received fresh medium containing MTT at 1 mg/mL. The plates were wrapped in foil and incubated for 4 h at 37 °C. After that, the medium containing the MTT was removed, and the resulting formazan salt was solubilized in DMSO. The absorbance was quantified at 620 nm using an absorption spectrometer (Synergy™ H1, Berlin, Germany).

### 2.11. Cell Cycle Analysis

A total of 2 × 10^5^ Jurkat T cells/well was seeded in 6-well plates and incubated using the intestinal digests diluted to 10% in cell media (C; G; A; A + G) for 7 days (representing 0.7 µM AFB1 in the cell media of A and A + G exposures), with the experiment carried out in quadruplicate. The cell suspension was poured into a 15 mL falcon and centrifuged for 5 min at 300× *g* at room temperature. The supernatant was aspirated, leaving about 50 μL of residual fluid in the tube to avoid disturbing the pellet. The CycleTESTTM PLUS kit protocol was followed. Buffer solution (100 µL) was added to the cell pellet and resuspended by gentle vortexing at low speed. They were again centrifuged for 5 min at 300× *g* at room temperature, and this step was repeated two more times. Staining was conducted by using PI stain solution for 10 min in the refrigerator in the dark. Samples were analyzed by flow cytometry with the appropriate settings.

### 2.12. Apoptosis/Necrosis Pathway Analysis

Cells were exposed as explained in Section 2.11. Cells were washed with 200 µL of binding buffer and centrifuged at 300× *g* for 10 min. The supernatant was completely aspirated, and the cell pellet was resuspended in 200 μL of 1× binding buffer. Following the fabricant instructions, 10 µL of Annexin V–FITC was added to the cells. The samples were mixed and incubated for 15 min in the dark at room temperature. After centrifuging and aspirating the supernatant completely again, the cell pellet was resuspended in 200 µL of binding buffer. Finally, 5 μL of PI (100 μg/mL) solution was immediately added prior to analysis by flow cytometry.

### 2.13. ROS Analysis

Cells were exposed as explained in Section 2.11. The cell suspension was centrifuged in a 15 mL falcon (300× *g* for 5 min). H_2_DCFA (5 µM as the final concentration) was added, and cells were incubated for 20 min in the dark at 37 °C. The tubes were centrifuged at 300× *g* for 5 min, washed, and suspended in 300 μL of PBS. In this assay, tert-Butyl hydroperoxide (TBHP) (1 mM; 30 min) was used as the positive control.

### 2.14. Mitochondrial ROS Analysis

Cells were exposed as explained in Section 2.11. Cells were centrifuged in a 15 mL falcon (300× *g* for 5 min). MitoSOX Red reagent was added (1 mM as the final concentration) in 500 µL of fresh medium and incubated for 20 min in the dark at 37 °C. The tubes were centrifuged at 300× *g* for 5 min and resuspended in 300 µL of PBS.

### 2.15. Mitochondrial Mass Analysis

Cells were exposed as described in Section 2.11, but in triplicate. A stock solution of MitoTracker probe was prepared by using DMSO, obtaining a concentration of 100 µM. The tubes were centrifuged (300× *g* for 5 min) to obtain a cell pellet and the supernatant was aspirated. The cells were resuspended gently in prewarmed (37 °C) staining solution containing the MitoTracker^®^ (100 nM) for 20 min in the dark at 37 °C. After staining was completed, cells were centrifuged (300× *g* for 5 min) and resuspended in 300 μL of PBS.

### 2.16. Cytometer Settings

All flow cytometry assays were carried out using the MACSQuant 16 analyzer (Miltenyi Biotech GmbH, Bergisch Gladbach, Germany). The total number of events per sample was 20,000 in each experiment. Blue (488 nm), violet (405 nm), and red (640 nm) lasers were used. Cell cycle results were collected using a 579/34 PE B2 filter. The fluorescence results of Annexin V, MitoTrackerTM, and H_2_-DCFDA were collected using a 525/50 FITC B1 filter. Fluorescence results of MitoSOXTM were collected using a V4 channel with 615/20 filter.

### 2.17. Statistical Analysis of the Data

Data were expressed as mean ± SD of four independent experiments. By using Student’s *t*-test for paired samples in Excel 2016, statistical analysis of the results was performed. *p* ≤ 0.05 was considered statistically significant. Macs quantify version was the software used for flow cytometry assays.

## 3. Results

### 3.1. AFB1 Bioaccessibility

The data regarding the matrix-matched curves, linearity range, regression coefficients (r^2^), equations, and limits of detection and quantification obtained in HPLC-FLD for the maize flours, bread, and simulated digests are shown in Table 2.

The concentration of AFB1 in naturally contaminated maize flour was 151.9 ± 1.4 mg/kg for E1 and 197.1 ± 3.8 mg/kg for E2. After baking, the AFB1 concentrations obtained for breads in E1 were 1.6 ± 0.1 mg/kg and 1.7 ± 0.1 mg/kg for A and A + G, respectively. In E2, the AFB1 concentrations of breads were 96.4 ± 9.6 mg/kg and 102.7 ± 4.4 mg/kg for A and A + G, respectively. Neither bread C or G showed AF content in E1 or in E2. The AFB1 concentration in bread was not measured before baking since the flours used as ingredients for making the bread had already been analyzed (Section 2.5 and Section 2.6). Regarding the gastric digests, the mean AFB1 concentrations were 0.176 ± 0.005 µM (A) and 0.191 ± 0.005 µM (A + G) for E1 and 3.934 ± 0.823 µM (A) and 3.861 ± 0.006 µM (A + G) for E2. In the intestinal digests, the AFB1 concentrations were 0.181 ± 0.002 µM and 0.162 ± 0.001 µM in E1 and 8.621 ± 0.021 µM and 7.742 ± 0.031 µM in E2 for A and A + G, respectively.

**Figure 1:**
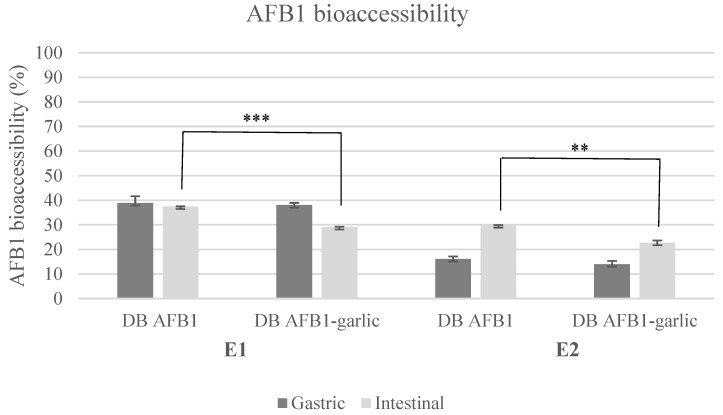
Bioaccessibility bar plot for experiments 1 and 2. Significant differences are indicated as *p* < 0.01 (**), *p* < 0.001 (***). E1, experiment 1. E2, experiment 2. DB, bread digest. Mean ± standard deviation (*n* = 3).

AFB1 bioaccessibility ranged from 29% to 39% in E1 and from 14% to 30% in E2 (Figure 1). Regarding E1, AFB1 bioaccessibility was 39 ± 3% (A) and 38 ± 1% (A + G) for the gastric digest and 37 ± 1% (A) and 29 ± 1% (A + G) for the intestinal digest. In E2, the results were 16 ± 1% (A) and 14 ± 1% (A + G) for the gastric digest and 30 ± 1% (A) and 23 ± 1% (A + G) for the intestinal digest.

### 3.2. Cell Viability

To evaluate the cell viability, an MTT assay was performed. Cells exposed to control bread intestinal digests were considered to have 100% viability for the calculations. No differences were observed between non-exposed cells and control cells. The addition of garlic (A + G) in contrast to the AFB1 intestinal digest (A) significantly increased the cell viability at 24 h in non-diluted digests and at 48 h (10–13%) and 72 h (11–18%) in all dilutions tested. Table 3 presents the cell viability percentages and statistical analysis of cells exposed to A + G and A intestinal digests.

### 3.3. Flow Cytometry Analysis

#### 3.3.1. Cell Cycle

**Figure 2:**
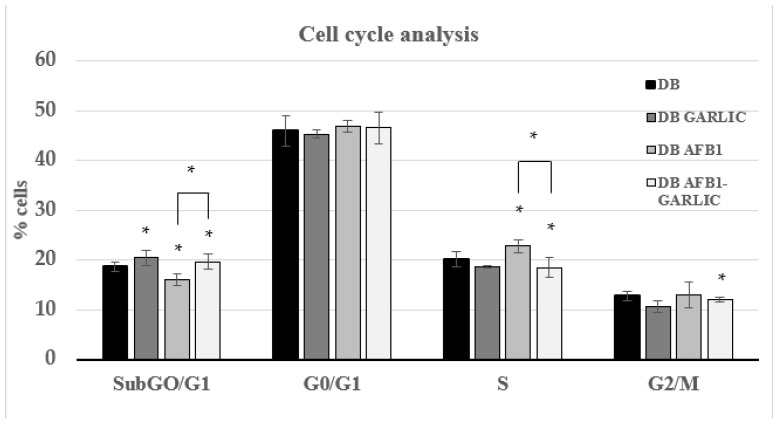
Effect of intestinal digests on Jurkat T cell cycle as assessed by flow cytometry. Cells were exposed for 7 days to bread digest (DB); garlic bread digest (garlic DB); AFB1 bread digest (0.7 µM) (AFB1 DB); and AFB1 (0.7 µM) + garlic (AFB1–garlic DB) bread digest. AFB1 concentration after both AFB1 and AFB1 + garlic exposures was 0.7 µM. *p* < 0.05 (*). Mean ± standard deviation (*n* = 4).

To evaluate the impact of the different digest breads on the Jurkat T cell cycle, PI dye was used. As Figure 2 indicates, cell cycle analysis was performed for every condition during the different phases. For the four conditions (C, G, A, A + G), at every cell phase, the results were, respectively: 19, 19, 16, and 20% for the Sub-G0/G1 phase; 46, 46, 47, and 47% for G0/G1; 20, 19, 23, and 19% for S; and 13, 12, 13, and 12% for G2/M.

#### 3.3.2. Apoptosis/Necrosis after Intestinal Digest Exposure

**Figure 3:**
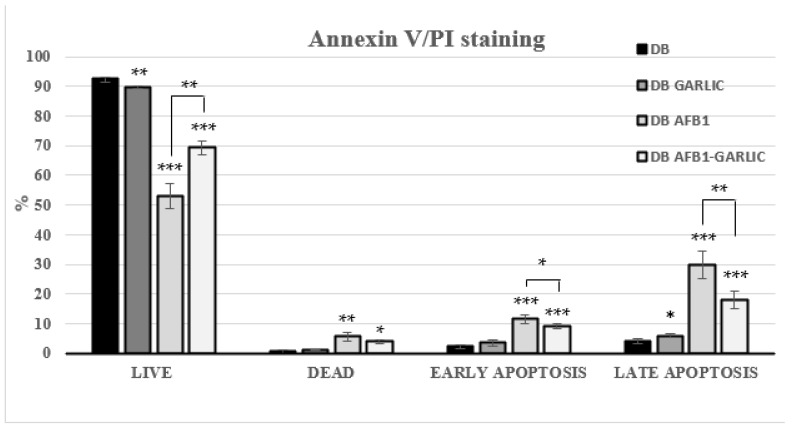
Effect of bread intestinal digest on apoptosis/necrosis as assessed by flow cytometry. Significant differences from the control, indicated by Jurkat T cells, compared with samples exposed for 7 days to bread digest (DB); bread digest garlic (garlic DB); AFB1 (0.7 µM) bread digest; and AFB1 (0.7 µM) + garlic bread digest. *p* < 0.05 (*), *p* < 0.01 (**), *p* < 0.001 (***). Mean ± standard deviation (*n* = 4).

Flow cytometry was used to assess the impact of the different bread digests on apoptosis/necrosis processes. Living cells accounted for 93% for C, 90% for G, 53% for A, and 69% for A + G; dead ones 1, 1, 6, and 4%; early apoptosis ones 2, 4, 12, and 9%; and late apoptosis ones 4, 6, 30, and 18%, respectively (Figure 3). A + G exposure significantly increased the live cell percentage and reduced early, and late apoptotic Jurkat T cells compared with A exposure. Annexin V−/PI− (live), Annexin V+/PI− (early apoptosis), Annexin V−/PI+ (necrosis), and Annexin V+/PI+ (late apoptosis) populations are shown in Figure 3.

#### 3.3.3. Effect of Intestinal Digest Exposure on ROS

**Figure 4:**
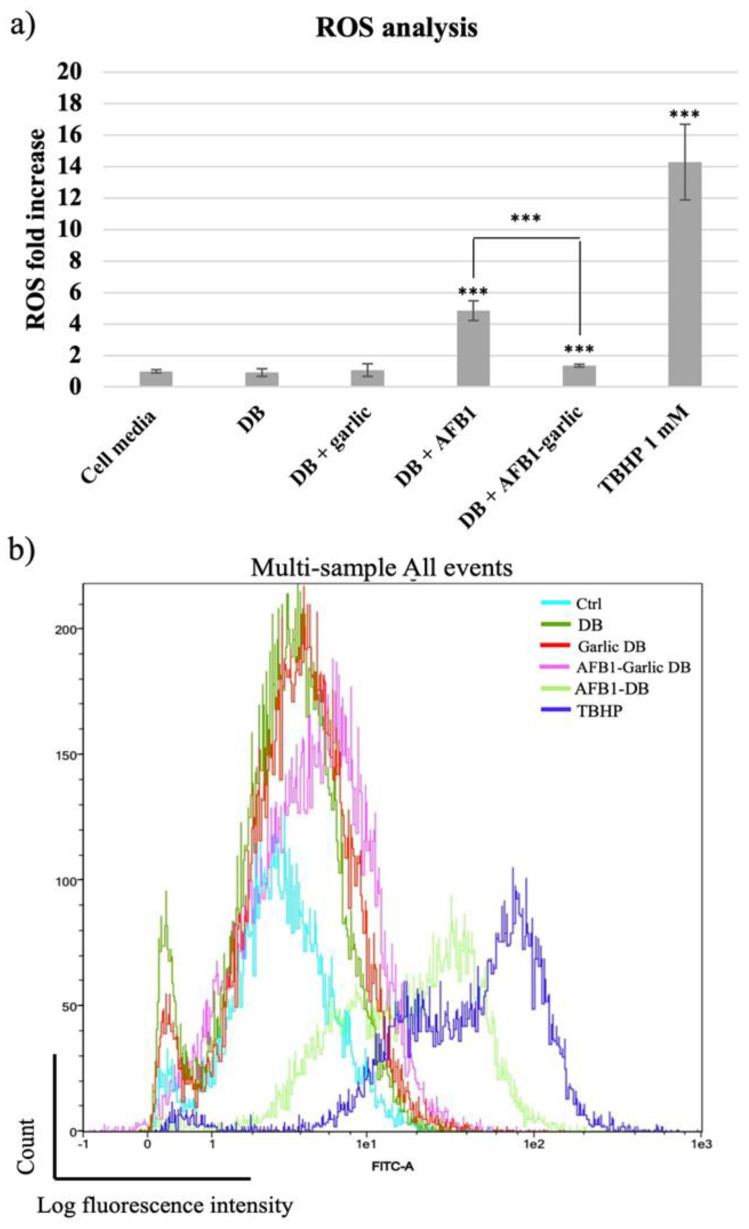
(**a**) Bar plot showing ROS production in Jurkat T cells exposed for 7 days to cell media, bread digest (DB); garlic DB; AFB1 DB; and AFB1–garlic DB in relation to DB. AFB1 concentration in both AFB1 DB and AFB1–garlic DB conditions was 0.7 µM. (**b**) Fluorescence representation obtained by overlaying the single plot of each condition by flow cytometry. ROS: reactive oxygen species; TBHP: tert-Butyl hydroperoxide. *p* < 0.001 (***). Mean ± standard deviation (*n* = 4).

From the lowest to the highest value, the control condition as well as the garlic condition revealed a 1-fold increase (FI) in ROS, while the AFB1–garlic condition showed a 2 FI, the AFB1 condition a 5 FI, and 1 mM THBP (a compound which enhances ROS production and was used as a positive control) revealed a 14.3 FI. The results indicate that AFB1 contributes to an increase in ROS production, while the presence of garlic diminishes the AFB1 effect (A + G) (Figure 4a,b).

**Figure 5:**
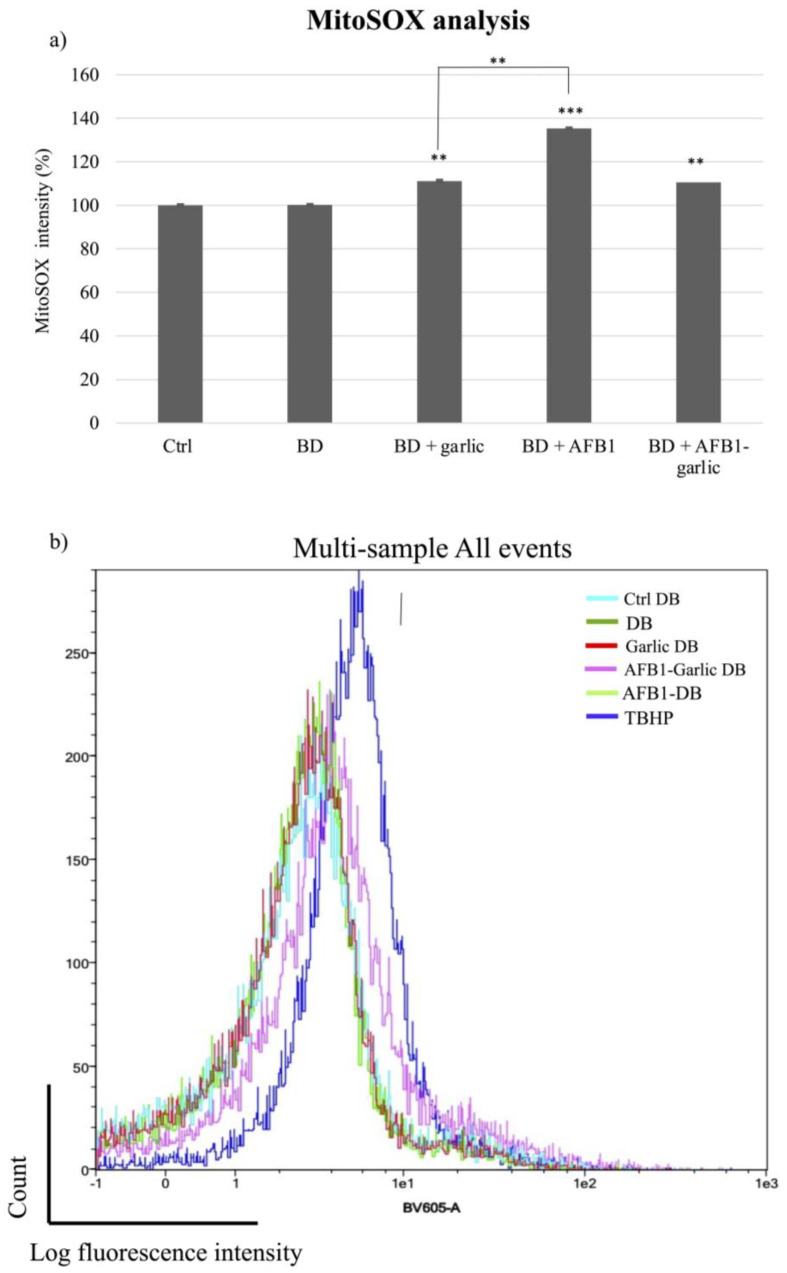
Effect of intestinal bread digests (0.7 µM) compared with control on mitochondrial ROS as assessed by flow cytometry. Significant differences from the control indicated as *p* < 0.01 (**), *p* < 0.001 (***). (**a**) Bar plots showing Jurkat T cells exposed for 7 days to control (non-exposed cells), digested bread (DB), garlic DB, AFB1 DB, AFB1 + garlic DB, and the negative control, and then stained with MitoSOX for flow cytometry detection. AFB1 concentration for both A and A + G exposures was 0.7 µM. (**b**) Fluorescence representation obtained by overlaying the single plot of each condition by flow cytometry. Mean ± standard deviation (*n* = 4).

To detect mitochondrial superoxide production in Jurkat T cells after exposures, MitoSOX Red dye was used. As Figure 5a,b indicate, the mitochondrial ROS analysis was normalized when the bread digest condition (control) showed 100% intensity. From the lowest to the highest value, the AFB1–garlic condition showed 111% intensity, the garlic condition evidenced 117%, and AFB1 condition revealed 135%. The results show that the presence of AFB1 enhances the production of ROS at a mitochondrial level (A), while the addition of garlic attenuates this effect (A + G).

#### 3.3.4. Mitochondrial Mass

**Figure 6:**
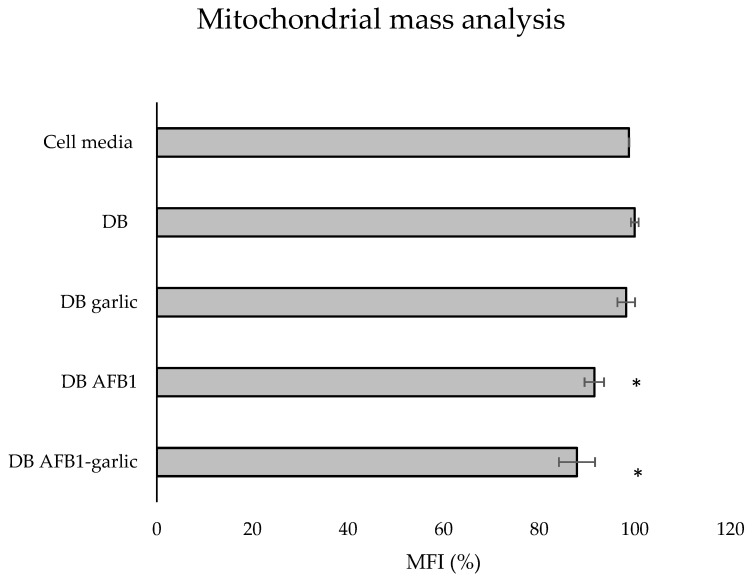
Effect of intestinal bread digests (0.7 µM) compared with control on mitochondrial mass as assessed by flow cytometry. Significant differences from the control indicated as *p* < 0.05 (*). Jurkat T cells were exposed to each exposure for 7 days (non-exposed cells), digested bread (DB), garlic DB, AFB1 DB, AFB1–garlic DB, and then fixed and stained with MitoTracker^®^ for flow cytometry detection. AFB1 concentration for both AFB1 DB and AFB1–garlic DB exposures was 0.7 µM. MFI: median fluorescent intensity. Mean ± standard deviation (*n* = 3).

To evaluate the effects of the different digests on the mitochondrial mass, MitoTracker^®^ dye was used. As shown in Figure 6, the bars indicate the results of each exposure, which stem from taking as a reference the C exposure as 100%. The A + G condition showed the lowest value (88%), followed by A (92%) and G (98%), while the C one revealed the highest mitochondrial mass (100%).

## 4. Discussion

In the last few years, AFB1 bioaccessibility has been studied in different food matrices by including functional ingredients. The inclusion of functional ingredients as a strategy to reduce exposure to AFB1 has offered promising results. Escrivá et al. [12] measured AFB1 bioaccessibility by using AFB1-contaminated wheat flour in the bread recipe, achieving concentrations of 78–164 µg AFB1/kg bread with and without 1% goat milk whey, fermented whey, or lyophilized pumpkin. After performing simulated human digestion, the intestinal AFB1 bioaccessibility was reduced by 64% when including 1% goat milk whey in the bread recipe, 57% with 1% fermented whey, and 74% with 1% lyophilized pumpkin. In this work, the intestinal AFB1 bioaccessibility reduction when including garlic was 7–8%, which may seem small compared with the reduction reported by Escrivá et al. [12]. Nevertheless, the total AFB1 bioaccessible fraction when including functional ingredients was smaller in this work, which used garlic. The main difference between these two studies is the AFB1 bioaccessibility result for AFB1-contaminated bread without functional ingredients. For Escrivá et al. [12], the result was 114%, while in this work, it was 38–29%. This may be caused by the different AFB1 concentrations used.

Other bioaccessibility studies have used AFB1 fortification with commercial AFB1 standards, including integrating mycotoxin in the food product after its production instead of simulating natural fungal contamination of the raw ingredients. It has been reported that fortification enhances the release of bioactive compounds [14]. In the only published article comparable to this work, Llorens et al. [15] found intestinal AFB1 bioaccessibility values like the ones found in this study for both bread with the addition of red beetroot (10–45%) and bread without this addition.

Cell viability studies exposing differentiated Caco-2 cells to AFB1 have been conducted [20,21]. Gao et al. [24], by using the CCK-8 assay, showed that Caco-2 cell exposure to AFB1 (1.6–25.6 µM) led to a cell viability decrease in a dose-dependent manner at 48 h. Zhang et al. [25] showed that Caco-2 cell exposure to 0.032–3.2 µM AFB1 also decreased cell viability in a dose-dependent manner by using the MTT technique. In accordance with the literature, the present study also showed a cell viability decrease due to the exposure to AFB1. Not only are the techniques used different, except for Zhang et al. [25], but the mycotoxin AFB1 concentration and time also varied.

The protective effects of garlic on differentiated Caco-2 cells have been studied previously. Engdal and Nilsen [26] showed that natto K2 and green tea were characterized by their ability to increase cell viability in Caco-2 cells, while garlic had no significant effects (0.017–0.17% *p*/*v*) after 90 min of exposure. Eguchi et al. [27] revealed that that aqueous garlic extracts decreased cell viability to <35% after 24 h of exposure at 5–25% (*v*/*v*). The sample used for cell exposure was the supernatant, while the pellet, which might still contain a considerable fraction of garlic, was discarded. In this experiment, the Voghiera garlic added as a functional ingredient to bread underwent a cooking process and an *in vitro* digestion. The garlic concentration in the bread was 2% (*w*/*v*), but after the *in vitro* digestion and media dilution, it was 0.002% (*w*/*v*). According to Bhatt and Patel [28], while cooking drastically reduces the total amount of phenolic compounds, *in vitro* gastrointestinal digestion provides garlic extracts with much higher antioxidant potential than can be obtained using organic solvents.

The toxic effects triggered by AFB1 on cells and tissues are principally provoked through the inhibition of cell proliferation and cell cycle arrest, apoptosis, oxidative stress, endoplasmic reticulum stress, and autophagy [29]. Huang et al. [30] showed that in the IRM-32 neuroblastoma cell line, AFB1 induced significant S-phase arrest at concentrations of 6.4 µM and 19.2 μM for 24 h and 48 h. Zhu et al. [31] revealed S-phase arrest in HepG2 cells exposed to 0–48 µM AFB1 for 24 h; the arrest was especially significant at 32 and 48 µM AFB1. Liu et al. [32] showed that AFB1 exposure induced G0/G1 arrest in F344 rat hepatocytes when administering 100 or 200 μg/kg AFB1 for 28 days. In accordance with these previous studies, this study showed that AFB1 exposure can promote a possible arrest of the cell cycle in the S phase, while the addition of garlic decreases the S phase (23 vs. 19%), like the control, by significantly limiting eventual cell cycle arrest.

Regarding apoptosis, 3D4/21 cells’ apoptosis rate increased in a AFB1-dose-dependent manner [33]. In accordance with these results, this work found that exposure to AFB1 increases the number of apoptotic and necrotic cells, while the addition of garlic attenuates its effects. Comparing garlic vs. AFB1 and garlic exposures, the percentage of apoptotic cells reached its peak in the AFB1 one (41%), including cells in early and late apoptosis, while there was a decrease in the combined AFB1 and garlic (27%) sample (Figure 3). It was clearly shown that AFB1 enhances cell apoptosis, and that garlic attenuates AFB1-promoting apoptosis in Jurkat T cells.

Liu and Wang [34] showed that in primary broiler hepatocytes, exposure of samples to different AFB1 concentrations (0.5–5 µM) for 6 h led to significant ROS generation in a concentration-dependent manner. Wang, Xu, Yu, and Xu [35] also revealed that exposing broiler cardiomyocytes to different AFB1 concentrations (0.5–5 µM) for 12 h resulted in a significant increase in intracellular ROS production. Furthermore, it was observed that *Cetraria islandica* methanol extract caused an increase in the activity of superoxide dismutase (SOD) and glutathione peroxidase (GPx) and a decrease in MDA levels in human lymphocytes *in vitro* [36]. The protective effect of garlic regarding oxidative processes has also been documented. Not only does garlic contribute to the decrease in ROS levels, but it also mitigates other proinflammatory parameters, such as SOD, catalase (CAT), and GPx activities [37,38,39,40]. In accordance with the previous literature, this work showed that AFB1 increased ROS production five times in comparison to the control, while the addition of garlic attenuated the effect of AFB1, resulting in similar values to the control. This study confirms the capacity of garlic to lower ROS via AFB1 in Jurkat T cells for the first time.

Research on AFB1′s impact, specifically on mitochondrial ROS, is scarce in the literature. However, there are other mitochondrial parameters, such as ATP production, mitochondrial membrane permeability, and respiratory capacity, which may be good indicators of ROS production status [41]. Chen et al. [42] revealed that AFB1 reduced mitochondrial respiratory capacity and ATP production in HepG2 and Caco-2 cells, which are related to cell dysfunctions. Liu et al. [41] showed that AFB1 increased mitochondrial membrane permeability, which would result in a release of proteins such as cytochrome c to activate the caspase cascade and programmed cell death. Similarly, the present study revealed that AFB1 exposure increased mitochondrial ROS production (by 35%) when compared with AFB1 and garlic one (11% more), confirming the antioxidant effect of garlic against AFB1 at the mitochondrial level also (Figure 4).

Mitochondrial changes in response to different stimuli can be assessed by using mitochondrial dyes to track mitochondrial mass and volume [43]. AFB1 has been revealed to diminish mitochondrial mass in fish exposed to 5–10 µM AFB1 for 7–8 days and mice fed with 0.375–1.5 mg/kg AFB1 for 30 days [44,45]. However, no significant differences were found in Jurkat T cells in this study, suggesting that neither AFB1 nor garlic can affect the mass of this organelle (Figure 6). Overall, these results confirm the toxicity *in vitro* of AFB1 at very low concentrations and long exposure times.

## 5. Conclusions

Garlic’s presence contributed to reduced AFB1 bioaccessibility in bread (7–8%). Garlic was also revealed to increase cell viability (9–18%). Flow cytometry showed that garlic mitigates the toxic effects of AFB1 in Jurkat T-cells on the cell cycle by preventing the arrest of the S phase, on apoptosis/necrosis, and on cellular and mitochondrial ROS production. Therefore, the antigenotoxic, antiapoptotic, and antioxidant effects of garlic were demonstrated. Nevertheless, further investigations are needed to confirm the protective role of garlic *in vivo* and its probable application in the food industry as prebiotic to counteract possible AFB1 food contamination at low levels and potential human toxicity.

## Figures and Tables

**Table 1:** Bread composition in experiments 1 and 2.

Ingredient (g)	C		G		A		A + G	
	E1	E2	E1	E2	E1	E2	E1	E2
Wheat flour	127	127	124	124	121.1	72.3	118.1	69.3
C maize flour	-	-	-	-	5.9	54.7	5.9	54.7
Water	66	66	65	65	66	66	65	65
Salt	2.6	2.6	2.6	2.6	2.6	2.6	2.6	2.6
Sugar	4	4	4	4	4	4	4	4
Fresh yeast	8	8	8	8	8	8	8	8
Garlic	-	-	4	4	-	-	4	4
Total quantity	207.6	207.6	207.6	207.6	207.6	207.6	207.6	207.6

C (control). E1 (experiment 1). E2 (experiment 2). (G) garlic. A (Aflatoxin B1). A + G (Aflatoxin B1 + garlic). C (contaminated). - (no content) (*n* = 3).

**Table 2:** Validation parameters for AFB1 quantitative determination method.

Matrices	Linearity Range	r^2^	Linear Regression Equation	LOD/LOQ (µg/L)
Maize flour	0.05–5 mg/L	0.9998	y = 516.44x − 4.7063	0.25/0.75
Bread	0.05–0.5 mg/L	0.9948	y = 389.03 − 3.1217	0.25/0.75
	0.05–5 mg/L	0.9999	y = 400.28x − 9.6478	0.25/0.75
Gastric digest	5–200 µg/L	0.9998	y = 0.7611x − 0.3598	0.25/0.75
	50–1500 µg/L	0.9996	y = 1134.4x − 14.942	0.25/0.75
Intestinal digest	5–50 µg/L	0.9984	y = 0.8279x − 0.7944	0.25/0.75
	10–1500 µg/L	0.9994	y = 633.28x − 4.3939	0.25/0.75

LOD, limit of detection. LOQ, limit of quantification. r^2^, regression coefficient. Mean ± standard deviation (*n* = 4).

**Table 3:** Cell viability of AFB1 and AFB1 + garlic samples at different times and dilutions. Cell viability (%) results obtained for differentiated Caco-2 cells after exposure to AFB1 and AFB1–garlic bread intestinal digests.

Cell Viability (%)						
Exposure Time	24 h		48 h		72 h	
Intestinal Digest	*AFB1*	*AFB1–Garlic*	*AFB1*	*AFB1–Garlic*	*AFB1*	*AFB1–Garlic*
1/32	79 ± 4	86 ± 2	74 ± 4	86 ± 4 *	73 ± 1	87 ± 6 ***
1/16	76 ± 5	84 ± 6	72 ± 5	85 ± 4 *	72 ± 9	83 ± 2 **
1/8	76 ± 7	81 ± 3	72 ± 1	83 ± 5 **	66 ± 2	83 ± 3 *
1/4	76 ± 3	80 ± 2	69 ± 4	80 ± 3 *	64 ± 8	82 ± 7 ***
1/2	73 ± 6	79 ± 7	68 ± 4	78 ± 4 **	63 ± 6	81 ± 3 *
*No dilution*	70 ± 4	79 ± 6 *	66 ± 5	77 ± 4 **	62 ± 4	79 ± 3 *

*p* ≤ 0.05 (*); *p* ≤ 0.01 (**); *p* ≤ 0.001 (***). Mean ± standard deviation (*n* = 4).

## Data Availability

Data are contained in the article.
